# A field trial of spinosad for the treatment and prevention of flea infestation in shepherd dogs living in close proximity to flea-infested sheep

**DOI:** 10.1186/s13071-015-0945-8

**Published:** 2015-06-12

**Authors:** Manolis N. Saridomichelakis, Manolis K. Chatzis, Theodoros Petanides, Elias Papadopoulos

**Affiliations:** Clinic of Medicine, Faculty of Veterinary Science, University of Thessaly, 224 Trikalon Str., GR-43100 Karditsa, Greece; Laboratory of Parasitology and Parasitic Diseases, Faculty of Veterinary Sciences, Aristotle University of Thessaloniki, University Campus, GR-54124 Thessaloniki, Greece

**Keywords:** Canine, *Ctenocephalides canis*, *Ctenocephalides felis*, Fleas, Insecticide, Livestock, *Pulex irritans*

## Abstract

**Background:**

Three flea species, *Pulex irritans*, *Ctenocephalides canis* and *C. felis* parasitize shepherd dogs living on sheep farms in Greece. The aim of this randomized, blinded, placebo-controlled trial was to examine the efficacy of spinosad, when administered three times every 4 weeks, as the only intervention to treat and prevent flea infestations in shepherd dogs living on sheep farms.

**Methods:**

Thirty dogs living on sheep farms and infested by at least 24 fleas were randomly allocated into equal groups. Group A dogs received spinosad (45–70 mg/kg body weight), every 4 weeks for three administrations, whereas Group B dogs were placebo-treated. Flea counting was performed at the beginning of the trial (day 0) and after 14, 28, 56 and 84 days. The first five fleas from each dog and 2–6 fleas collected from 5–11 sheep were used for species identification.

**Results:**

The percentage of dogs with zero flea counts was significantly higher in group A than in group B at days 14, 28, 56 and 84 and flea counts were significantly lower in group A than in group B at days 14, 28, 56 and 84. In group A, flea counts were significantly lower at days 14, 28, 56 and 84 compared to day 0 whereas there were no changes in flea counts of group B dogs. The percent efficacy of spinosad for the treatment and prevention of flea infestation was higher than 98 % (arithmetic means) or higher than 99 % (geometric means) throughout the study. No adverse reactions were recorded.

*C. canis* was the predominant flea species of dogs at day 0. In group A the relative abundance of *C. felis* increased at day14 whereas in group B the relative abundance of *P. irritans* increased at days 14, 28, 56 and 84.

**Conclusions:**

Spinosad is safe and effective for the treatment of *C. canis* and *C. felis* infestations and for the prevention of *P. irritans*, *C. canis* and *C. felis* infestations in shepherd dogs living in close proximity to sheep.

## Background

In many areas of the word, fleas are common ectoparasites of dogs [1]. They can cause blood loss anemia, flea bite and flea allergic dermatitis and they are intermediate hosts of parasites and vectors of bacterial pathogens of zoonotic importance, such as *Bartonella* spp. [1, 2]. *Ctenocephalides felis* is generally considered to be the most common flea species parasitizing dogs [1]. However, in Greece, *C. canis* has been found to be more prevalent than *C. felis* among dogs admitted to a University Teaching Hospital in the northern part of the country [3]. More recently, when dogs living on dairy goat and sheep farms located in central and southern Greece were examined, the most common flea species found was *Pulex irritans*, followed by *C. canis* and then by *C. felis* [4]. Also, a high flea burden has been noticed in some dogs living on sheep farms in central Greece that have been admitted, for various reasons, to the author’s University Clinic, probably because no anti-flea interventions had been used on the dogs, the sheep or the environment (unpublished observations).

Spinosad is a mixture of spinosyns A and D that, after ingestion by the insect during blood meal, targets their nicotinic acetylcholine receptors and gamma-aminobutyric acid neurotransmission, leading to hyperexcitation and death [5–7]. When administered orally, spinosad is effective for the treatment of pre-existing and for the prevention of new infestations by *C. felis* and *C. canis* under laboratory conditions [5, 6, 8–12], by *C. felis* in a simulated home environment [13] and by undetermined flea species under field conditions [5, 14, 15] for up to 4 weeks.

The aim of this randomized, blinded, placebo-controlled trial was to examine the efficacy of spinosad, when given every 4 weeks for 3 consecutive administrations, as the only intervention to treat and prevent flea infestation in shepherd dogs living on sheep farms in central Greece.

## Methods

### Ethical approval

The experimental protocol was in accordance with the Greek laws (1197/81 and 2015/92) and had been approved, on both legal and ethical grounds, by State authorities (license No 2537). Signed informed consent was obtained from dog/farm owners before enrollment of the dogs into the study.

In addition to the 1 h post-administration observation of all dogs by one of the investigators (MKC), owners were instructed to monitor each dog for the duration of the study and to report any possible adverse events whether or not considered to be treatment-related.

### Study dogs

A total of 30 flea-infested dogs living on sheep farms in central Greece were enrolled in this study. To be included in the study they should present no abnormalities on physical examination (including skin lesions indicative of flea allergic dermatitis like hypotrichosis, alopecia, crusts, excoriations, hyperpigmentation, lichenification in the posterior part of the body), lived on the same farm with at least one more dog eligible for the study, had been infested by at least 10 fleas at the beginning of the trial (day 0) and they should not have been treated with ectoparasiticides, including pyrethroid-impregnated collars, for a minimum time period determined on the product label (e.g. at least 8 months before enrollment for flumethrin-impregnated collars and 1 month for spot-ons labeled for monthly use). Dogs younger than 14 weeks of age, with a body weight of less than 3.9 kg, with pre-existing diseases, under extra-label treatment with ivermectin for demodectic mange or other ectoparasitoses [7, 16, 17], as well as pregnant or lactating females were excluded from the study.

### Study groups

**Fig. 1:**
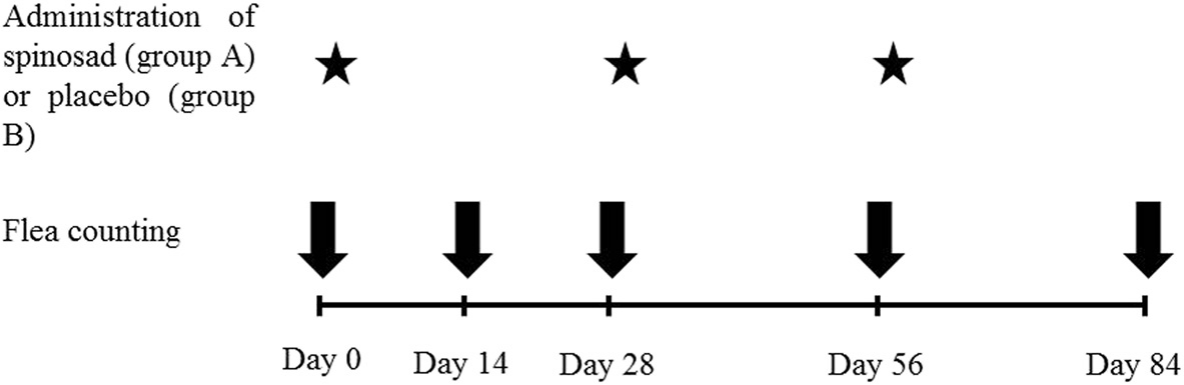
Design of the study. Time points of flea counting and administration of spinosad (group A dogs) or placebo (group B dogs)

The dogs were randomly allocated into two groups (group A and group B). For this purpose, all eligible dogs living on each farm were considered as a block and per block randomization was done using a random number generator software, freely available from the internet (http://www.random.org/). Group A dogs (n = 15) received spinosad (Comfortis; Elanco Animal Health), at the dose registered in Europe (45–70 mg/kg body weight) [5], every 4 weeks for three administrations (Fig. 1). Spinosad was given with food by the owner, under the supervision of a member of the research team (MKC) who monitored the dogs for the next 1 h; if vomiting or regurgitation occurred during this period spinosad administration had to be repeated. Group B dogs (n = 15) received placebo tablets made of starch and inert excipients that were administered in the same way and at the same time intervals as spinosad in group A dogs (Fig. 1). The trial was conducted from April to July 2014 and no other ectoparasiticides were used on the dogs, the sheep or the environment.

### Flea counting and identification

Flea counts were performed at day 0 and after 14, 28, 56 and 84 days (Fig. 1) by another investigator (TP) who was blinded as to each dog’s group. Dogs were separated from the flock at least 4 h before (the exact time was not recorded but it is estimated to range from 4 to 6 h) and were kept in a near-by open area where flea counting was performed before the administration of spinosad or placebo. Each dog was combed all over the body with a fine-tooth flea comb for at least 10 min and until no fleas could be recovered for the last minute and all live fleas were captured and counted. The first five fleas were placed in a separate vial with 90 % ethanol for species identification, whereas the remaining fleas were temporarily kept in a zip-closing plastic bag and were returned to the back of the dog at the end of the procedure. At the same time points, 2–6 fleas were collected manually from each of 5–11 randomly selected sheep and were placed in separate vials with 90 % ethanol for species identification. Species identification of fleas was based on microscopic examination as described by Wall and Shearer [18].

### Statistical analysis

The sex, age and body weight of the dogs as well as the number of fleas per dog at day 0 were compared among the different farms with Fisher’s exact test (sex) and one-way ANOVA (age, body weight, number of fleas). Also, the two groups of dogs were compared in terms of their distribution among the different farms (Fisher’s exact test) and the sex (*χ*^2^ test), age, body weight and number of fleas per dog at day 0 (independent samples *t*-test).

The geometric mean number of fleas for each group of dogs at the different time points of the study was calculated after adding 1 to the flea count of each dog, calculating the natural logarithm of (flea count +1), calculating the arithmetic mean of these logarithms, calculating the antilogarithm of the arithmetic mean and subtracting 1 from the antilogarithm. The percent efficacy of spinosad was calculated at the different time points of the study, using both the geometric and the arithmetic means of flea counts, with the formula $E=\frac{Mc-Mt}{Mc} \times 100$, where E is the percent efficacy, Mc the mean (geometric or arithmetic) flea count in the controls (group B) and Mt the mean (geometric or arithmetic) flea count in the spinosad-treated dogs (group A).

The number of dogs with zero flea counts and the flea counts at the different time points of the study was compared between the two groups with *χ*^2^ test and with independent samples *t*-test, respectively. At 5 % level of significance, the power of the study was 80 % to detect a 54 % difference between the two groups in the number of dogs with zero flea counts and 80 % to detect a 21 % difference of flea counts between the two groups, assuming a standard deviation of 0.2.

For each group of dogs, flea counts were compared among all time points of the study with Friedman’s two-way ANOVA; when a significant difference was found post-hoc tests (related samples Wilcoxon Signed Rank test) were used to examine for differences between all pairs of time points.

The relative abundance of each flea species was calculated as it’s percentage among all fleas identified, separately for group A dogs, for group B dogs and for sheep. The relative abundance was compared among the farms at each time point of the study, for each farm separately among the time points of the study, and, after combining the results from all farms, among the time points of the study with Fisher’s exact test.

Statistical analysis was done using SPSS 20 for Windows and the level of significance was set at 5 %.

## Results

### Dogs, group allocation, treatment administration and adverse events

A total of 30 dogs, living on three different sheep farms (designated as farms A, B and C) were screened and all of them were eligible for the study and they completed the 84 day trial. In farm A there were 10 dogs and 30 sheep, in farm B 16 dogs and 43 sheep and in farm C 4 dogs and 21 sheep (Table 1). All dogs lived mainly outdoors and had free access to and close contact with sheep. All 30 dogs were mongrels, they included 16 (53.3 %) intact males and 14 (46.7 %) intact females, their age ranged from 1 to 7 years (mean ± standard deviation: 3.15 ± 1.65 years) and their body weight ranged from 12–50 kg (mean ± standard deviation: 28.53 ± 11.13 kg).

**Table 1:** Description of the three farms

	Farm A	Farm B	Farm C
Number of sheep	30	43	21
Number of dogs	10	16	4
Number (%) of group A dogs	6 (60 %)	8 (50 %)	1 (25 %)
Spinosad dose (mean ± SD) in mg/kg	57 ± 8.84	55.1 ± 6.89	69.33
Number (%) of group B dogs	4 (40 %)	8 (50 %)	3 (75 %)

Number of sheep in the three farms, number of dogs and allocation of the dogs into group A (spinosad-treated) and group B (placebo-treated)

Fifteen dogs (6 from farm A, 8 from farm B and 1 from farm C) were allocated to group A (spinosad) and 15 dogs (4 from farm A, 8 from farm B and 3 from farm C) were allocated to group B (placebo) (Table 1). Spinosad dose for group A dogs ranged from 45 to 69.3 mg/kg body weight (mean ± standard deviation: 56.82 ± 8.04 mg/kg body weight). Flea counts at day 0 ranged from 24 to 73 fleas per dog (median: 48; arithmetic mean: 49.3; geometric mean: 47.1) in group A and from 31 to 76 fleas per dog (median: 56; arithmetic mean: 53; geometric mean: 51.1) in group B (Table 2). No significant differences were found in the distribution of the two groups of dogs among the 3 farms (P = 0.47) or in their sex (P = 1), age (P = 0.14), body weight (P = 0.73) and flea counts at day 0 (P = 0.48).

**Table 2:** Flea counts, number of dogs with zero flea count and percent efficacy of spinosad

	Day 0		Day 14		Day 28		Day 56		Day 84	
	Flea counts									
Group	A	B	A	B	A	B	A	B	A	B
Range	24-73	31-76	0-4	22-72	0	21-69	0	24-79	0	20-86
Median	48	56	0	46	0	56	0	53	0	64
Arithmetic mean	49.27	53	0.6	47	0	51.27	0	51.33	0	58.67
Geometric mean	47.14	51.05	0.25	44.34	0	48.82	0	48.38	0	54.57
	Number (%) of dogs with zero flea counts									
Group A (n = 15)	0 (0 %)		12 (80 %)		15 (100 %)		15 (100 %)		15 (100 %)	
Group B (n = 15)	0 (0 %)		0 (0 %)		0 (0 %)		0 (0 %)		0 (0 %)	
	Percent efficacy of spinosad									
Arithmetic mean			98.72 %		100 %		100 %		100 %	
Geometric mean			99.44 %		100 %		100 %		100 %	

Range, median, arithmetic and geometric means of flea counts of spinosad-treated (group A) and of placebo-treated (group B) dogs, number of group A and group B dogs with zero flea counts at the beginning of the trial (day 0) and after 14, 28, 56 and 84 days and percent efficacy of spinosad

All treatments were administered according to the study protocol and no vomiting or other adverse reactions were witnessed throughout the trial.

### Flea counts and efficacy of spinosad

Range, median, arithmetic and geometric means of flea counts of group A and group B dogs at the various time points of the trial, the number of dogs with zero flea counts and the percent efficacy of spinosad, based on both arithmetic and geometric mean of flea counts, are shown on Table 2.

The percentage of dogs with zero flea counts was significantly higher in group A than in group B at days 14 (80 % vs 0 %), 28 (100 % vs 0 %), 56 (100 % vs 0 %) and 84 (100 % vs 0 %; P < 0.001 for all comparisons) and flea counts were significantly lower in group A than in group B at days 14, 28, 56 and 84 (P < 0.001 for all comparisons). In group A, flea counts were significantly different among the five time points of the trial (P < 0.001) and post-hoc testing revealed that they were significantly lower at days 14 (arithmetic mean: 0.6, geometric mean: 0.25), 28, 56 and 84 (zero arithmetic and geometric means) compared to day 0 (P < 0.001 for all comparisons) without difference between any other time points. On the contrary, no significant difference was found in flea counts of group B dogs among the five time points of the trial (arithmetic means: 47–58.67, geometric means: 44.34-54.57; P = 0.07).

The percent efficacy of spinosad for the treatment of pre-existing and the prevention of newly acquired flea infestations, under the conditions of this trial, was higher than 98 % (arithmetic means) or higher than 99 % (geometric means) at 14, 28, 56 and 84 days (Table 2).

### Species of fleas parasitizing dogs and sheep

Fleas were found on group A (spinosad-treated) dogs at days 0 and 14 only (Table 2) and *C. canis* was predominant over *C. felis* (Table 3). The relative abundance of these two flea species did not differ among the three farms at either day 0 or 14, whereas, the relative abundance of *C. felis* was higher at day 14 compared to day 0 (P = 0.003).

**Table 3:** Flea species in group A and group B dogs

	Day 0	Day 14	Day 28	Day 56	Day 84
	Group A (spinosad)				
*Ctenocephalidescanis*	61/62 (98.4 %)	4/7 (57.1 %)	N/A	N/A	N/A
*Ctenocephalidesfelis*	1/62 (1.6 %)	3/7 (42.9 %)	N/A	N/A	N/A
*Pulexirritans*	0/62 (0 %)	0/7 (0 %)	N/A	N/A	N/A
	Group B (placebo)				
*Ctenocephalidescanis*	60/62 (96.8 %)	23/68 (33.8 %)	9/69 (13 %)	8/73 (11 %)	7/71 (9.9 %)
*Ctenocephalidesfelis*	2/62 (3.2 %)	2/68 (2.9 %)	0/69 (0 %)	0/73 (0 %)	0/71 (0 %)
*Pulexirritans*	0/62 (0 %)	43/68 (63.2 %)	60/69 (87 %)	65/73 (89 %)	64/71 (90.1 %)

Relative abundance of each flea species on spinosad-treated (group A) and placebo-treated (group B) dogs at the beginning of the trial (day 0) and after 14,28, 56 and 84 days

N/A: non-applicable (due to zero flea counts)

Fleas were found on group B (placebo-treated) dogs at all time points (Table 2). *C. canis* was the most prevalent flea species at day 0 but thereafter *P. irritans* predominated (Table 3). The relative abundance of *C. canis* was significantly higher at day 0 compared to days 14, 28, 56 and 84 and at day 14 compared to days 28, 56 and 84, whereas the opposite was found for *P. irritans* (P ≤ 0.002 for all comparisons). The only difference (P ≤ 0.001 for all comparisons) among the three farms was noticed at day 14 when only *P. irritans* was found on farm A dogs, both *P. irritans* (60.5 %) and *C. canis* were found on farm B dogs and both *C. canis* (80 %) and *C. felis* (20 %) were found on farm C dogs. The time that the relative abundance of *C. canis* and *P. irritans* changed also differed among the farms. In farm A the relative abundance of *C. canis* was higher and that of *P. irritans* was lower at day 0 compared to all other time points (P < 0.001 for all comparisons) but there were no differences among days 14, 28, 56 and 84 (i.e. significant changes occurred in the first 14 days). In farm B, in addition to the differences noticed in farm A (P < 0.001 for all comparisons), the relative abundance of *C. canis* was lower and that of *P. irritans* was higher also at day 14 compared to days 28, 56 and 84 (P ≤ 0.015) and there were no differences among days 28, 56 and 84 (i.e. significant changes occurred in the first 28 days). Finally, in farm C there was no difference between days 0 and 14, a significant decrease on *C. canis* and a significant increase of *P. irritans* relative abundance was found in the comparisons between day 0 and days 28, 56 and 84 (P < 0.001 for all comparison) as well as between day 14 and days 28, 56 and 84 (P < 0.001 for all comparison) (i.e. significant changes occurred between 14 and 28 days).

Fleas were found on sheep at all time points. *P. irritans* predominated (>88.9 %), followed by *C. canis* (0–18.2 %) and *C. felis* (0–5.6 %). There were no significant differences among the three farms but the comparison among time points showed increased prevalence of *C. canis* at day 28 (P = 0.037) and day 84 (P = 0.031) compared to day 0.

## Discussion

The high percent efficacy of spinosad for the treatment and prevention of flea infestations (>98 % and >99 % using arithmetic and geometric means, respectively) is comparable to the efficacy of this molecule that has been reported in most studies where dogs were experimentally infested by *C. canis* [10] or *C. felis* [5, 6, 9, 19], as well as after experimental infestation with *C. felis* of dogs in a home simulated environment [13]. Also, the reduction of flea counts witnessed in the present study was very similar to those reported previously in field trials of spinosad [5, 14, 15], spinosad-milbemycin oxime combination [20] and spinosad in combination with an amitraz collar [21]. Finally, the prevalence of spinosad-treated dogs with zero flea counts (80-100 %) is similar to that reported after experimental infestation by *C. felis* [19] or *C. canis* [10] and in field trials of spinosad alone [14] or spinosad-milbemycin oxime combination [20]. However, at least for some time points of this study, these figures seem to be higher compared to other field trials testing either spinosad alone [5] or spinosad in combination with an amitraz collar [21].

The separation of the dogs from the flock before flea counting as well as the duration of combing may have led to an overestimation of the percent efficacy of spinosad, of the prevalence of dogs with zero flea counts and/or of the flea count reductions in spinosad-treated dogs. All dogs were removed from the, presumably heavily infested, premises to a nearby open area for at least 4 h before flea counting in order to avoid, as much as possible, infestations by newly-emerged fleas. The 4 h time period was selected based on the results of experimental infestations showing that, due to the fast action of spinosad, a therapeutic efficacy of 80-100 % is anticipated at 4 h, at least for the first 2 weeks after drug administration and because this time is adequate for most newly acquired fleas to a blood meal [9, 10]. Although the separation from the flock is somehow similar to the movement of household dogs to a research facility for flea counting [14], it is logical to assume that spinosad-treated dogs experienced transient flea infestations during their normal daily routine in the farms. The duration of flea combing (at least 10 min and until no fleas were recovered for the last minute) used in the present study was in the range proposed by the recent guidelines of the World Association for the Advancement of Veterinary Parasitology [22]. However, it cannot be excluded that a lower prevalence of dogs with zero flea counts would have been recorded among spinosad-treated dogs if longer combing periods had been selected, like in some previous field trials [5, 14, 21].

On the other hand, the flea counts at the beginning of the present study (geometric mean in group A dogs: 47.14) was higher compared to most previously published field trials of spinosad or spinosad-milbemycin oxime combination (geometric means of spinosad treated dogs: 16.4 to 40.7) [5, 20, 21] and this is the only field study where a placebo group has been used. Furthermore, besides the placebo-treated group B dogs, there were additional untreated flea hosts (i.e. sheep) and no environmental control measures were applied. For these reasons, the >98 % efficacy and the 80-100 % prevalence of dogs with zero flea counts throughout this 3-month trial are particularly impressive.

Three flea species, *P. irritans*, *C. canis* and *C. felis*, were recovered from the sheep and there was a clear predominance of *P. irritans* throughout this trial whereas, in a previous study, *P. irritans* was the only species found in sheep living on dairy goat farms in central and southern Greece [4]. Surprisingly, *P. irritans* was not found in any group A or group B dog at day 0, whereas this species has been reported to predominate over *C. canis* and *C. felis* when 54 dogs living on dairy goat farms, with or without sheep, were examined [4]. This discrepancy may be explained by: a) the absence of goats, which are highly preferred hosts and heavily infested by *P. irritans* [4], in the three farms of our study; b) temporal differences (the previous study was conducted in June and July whereas the present study started in April); and c) the collection of only 5 fleas per dog (i.e. 6.6-20.1 % of the total flea burden) for species identification which was done in an effort to minimize interference with future flea counts [22]. Therefore, although it cannot be excluded that some dogs may have been infested by *P. irritans* at the beginning of the trial, it seems that *C. canis* shows a particular preference for these hosts.

There are two, non-mutually exclusive, explanations for the significant change in favor of *P. irritans* that was witnessed in placebo-treated (group B) dogs during this trial, namely a seasonal effect and an effect of spinosad treatment of group A dogs. At least in goats, the intensity of infestation by *P. irritans* has been shown to increase steadily from May to June [4] and if the same applies for the dogs it could explain the increasing relative abundance of this species from the beginning (April) towards the end (July) of the present study. However, the increased relative abundance of *C. canis* in sheep at days 28 and 84 does not seem to be in favor of this explanation. The fast adult flea killing activity of spinosad has a strong negative impact on egg production so that new adult flea emergence progressively declines when all dogs (and obviously other possible hosts) are treated [9, 12, 13]. In our study, administration of spinosad in some of the dogs in each farm may have reduced the relative abundance of *C. canis*, which was the predominant flea species at day 0, thus leading to an increased relative abundance of *P. irritans*. The observation that this change occurred earlier in farm A (60 % of the dogs received spinosad) than in farm B (50 % of the dogs received spinosad) and even later in farm C (25 % of the dogs received spinosad) is in favor of this explanation.

Vomiting is the most common adverse reaction of spinosad that has been recorded in both experimental studies and field trials [9, 19]. In the present study, neither vomiting nor any other adverse reactions were recorded, confirming the high tolerance of this molecule.

## Conclusions

When administered at 45–69.3 mg/kg body weight every 4 weeks for three times in shepherd dogs living in close proximity to sheep, the efficacy of spinosad for the treatment of infestations by at least two flea species (*C. canis* and *C. felis*) and for the prevention of infestations by *P. irritans*, *C. canis* and *C. felis* was 98.7 % (arithmetic means) or 99.4 % (geometric means) at 14 days and 100 % thereafter. The efficacy of this molecule is further supported by the number of treated dogs with zero flea counts (80 % at 14 days and 100 % thereafter). The results demonstrate that spinosad is safe and effective for the treatment and prevention of flea infestation in shepherd dogs living in close proximity to sheep.
